# Implications of Ethiopian Productive Safety Net Programme on household dietary diversity and women’s body mass index: a cross-sectional study

**DOI:** 10.29219/fnr.v62.1574

**Published:** 2018-11-01

**Authors:** Asnake Ararsa Irenso, Gudina Egata Atomsa

**Affiliations:** School of Public Health, Haramaya University, Harar, Ethiopia

**Keywords:** BMI, PSNP, social protection, HDD, Ethiopia, drought, women

## Abstract

**Introduction:**

Poor nutritional status of women remains a critical problem in Ethiopia. Nutrition for women matters not only for the public health relevance of breaking the intergenerational cycle of malnutrition but for its high return in other sectors such as education and health. The Ethiopian Productive Safety Net Programme (PSNP) is a program that protects chronically food-insecure households against food insecurity through cash or food transfer. However, its effect on food access and women’s body mass index (BMI) has remained unexplored.

**Objective:**

This study was intended to assess differences in household dietary diversity (HDD) and women’s BMI and associated factors among PSNP and non-PSNP households.

**Methods:**

This community-based cross-sectional study was carried out in the Kombolcha District of Eastern Ethiopia from July 1 to 28, 2015. HDD and women’s BMI were compared. Ordinal logistic regression was used to identify factors associated with women’s BMI.

**Result:**

The prevalence of undernutrition was 27.3% (95% confidence interval [CI]: 23.8–30.9) and 20.2% (95% CI: 17.1–23.5) for women from PSNP and non-PSNP households, respectively. PSNP membership had a significant effect on HDD and minimal effect on women’s BMI. Ordinal logistic regression yielded significant associations for medium wealth status, with an odds ratio (OR) of 0.533 (95% CI: 0.339–0.837), uptake of better health care services compared to previous year with an odds ratio (OR) of 0.647 (95% CI: 0.429–0.974) and reduction in selling assets for the sake of buying food with an OR of 1.575 (95% CI: 1.057–2.349).

**Conclusion and recommendation:**

There was high magnitude of chronic energy deficiency among PSNP and non-PSNP households, at 27.3 and 20.2%, respectively, and it was associated with economic status and health care utilization, suggesting the need to promote profitable income-generating activities and nudging for minimum health care as a condition for transfer.

Launched in 2005, the Ethiopian Productive Safety Net Programme (PSNP) is the largest African social protection program to provide predictable transfers. The government launched Phase 4 of the PSNP in 2015 (1). The main objective of the PSNP is to increase livelihoods and resilience to shocks and to improve food security and nutrition for rural households vulnerable to food insecurity. It has public works, direct support and livelihood components directed at heads of household. The program enables households with an able-bodied adult in public works to receive cash or in-kind transfers for 6 months of the year. By contrast, households without labor capacity become permanent, direct support clients; they receive 12 months of unconditional transfers and are linked with social protection services. Beneficiaries are paid in cash equivalent to 15 kg of cereals and 4 kg of pulses per month (adjusted for inflation). The wage rate used to compute the transfer in cash varies according to the purchasing power in different areas. The livelihood component includes skill-related trainings provided by appropriate institutions or conditional capacity building of grassroots institutions such as farmer training centers (1, 2).

Malnutrition has remained a major global issue in the post-Millennium Development Goals era. Ending this problem has continued to receive more attention and commitment for its extraordinary contribution towards the progress of Sustainable Development Goal (SDG) targets (3). This remains true in Ethiopia, where poor nutritional status of women remains a central problem. According to the Ethiopian Demographic and Health Survey (EDHS) (2), about 27% of women of reproductive age are chronically malnourished (4). Since 2016, the PSNP has been closely connected to the National Nutrition Programme and particularly the Community-Based Nutrition Programme, which focuses on nutrition education for mothers and supplementary food for children and mothers. In this regard, Ethiopia’s PSNP has a critical role in advancing and accelerating progress toward improved food and nutrition security mentioned under SDG 1 (5, 6).

Nutrition for women matters not only for the public health relevance of breaking the intergenerational cycle of malnutrition but also for its high return in numerous other sectors such as education and community health. A major current focus of women’s nutrition revolves around how to ensure improved decision-making for women and in turn a greater right to food and nutrition security, in addition to maximized improvement in social protection programs (7, 8). Social protection is a nutrition-sensitive development effort aimed at improving nutrition among nutritionally vulnerable populations and individuals (9).

Productive safety nets have a direct impact on consumption and a secondary impact on school attendance, enrolment and retention, utilization of health services, food security, nutrition, and accumulation of assets and building of livelihoods (10). The impetus of PSNP was to deliver a predictable package of support for a number of years that leads to asset accumulation and enable households to cover year-round food needs, in addition to building resilience to moderate shocks (state of graduation) (11). Though it has achieved this goal, improvements thought to achieve better nutrition and health outcomes have been implemented recently (1).

Ethiopia’s PSNP has improved nutritional outcomes by addressing the underlying causes of malnutrition (through nutrition-sensitive interventions) such as female empowerment, maternal and child health, infant and young child feeding practices, vaccination campaigns, dietary diversity, and water, sanitation, and hygiene. This work through soft conditionalities means public works clients have co-responsibilities to attend behavior change communication sessions for pregnant and lactating women (PLW) and their young children (1, 12). The program enhances household food consumption and dietary diversity by providing families with cash and increasing purchasing power. Transfers also work by minimizing negative coping mechanisms and empowering women in poverty to focus on specific services. This aids women in making better choices for self- and family care with an anticipated positive influence on the nutritional status of women and children (5, 13–15).

Ethiopia’s PSNP relies on meeting eligibility criteria that include being members of the community, having chronic household food insecurity, which is a food gap of 3 months or more per year, in the last 3 years; sudden food insecurity due to severe loss of assets and households with inadequate family support. Clients are selected through community-based targeting, meaning that a community food security task force facilitates client selection through reading client conditions out loud and discussing it against criteria at a public meeting (1). It aims to empower and support vulnerable women among other target groups by improving water security through pond construction or rehabilitation, improved firewood availability through rehabilitated natural environment and nutrition insecurity (16–18).

However, cascades of chronic food insecurity that lead to migration of key household members to towns for work leave women with an immense workload of home subsistence farming (normally more than 50% of women work in agriculture), childcare, and public work of PSNP (17, 19, 20). Provided unequal participation and benefit share from the PSNP, and high burden of agriculture, economic and domestic care work, women’s nutrition will continue to be a challenge (1). In addition, a cash-first principle of PSNP in male-headed households may lead to less control over transfer by women; males may misuse these funds, leading to conflicts. In other words, transfers not allocated to women influence intrahousehold resource allocation by leaving women with limited bargaining power. Despite the empowerment component in PSNP, a recent PSNP assessment has recognized women in male-headed and female-headed households as underserved and vulnerable groups (21–23). This is worsened by women’s triple burdens of land access; specifically, they account for less than 20% of the landholders, land policy that is based on state ownership of land (state controls land ownership, gives all rights except sale and mortgage) and limited agricultural inputs even when they have access to land (19, 24).

Based on the ethical notion of fairness and limited resources, the government of Ethiopia must use a fair mechanism for separating the population into those eligible and ineligible for social transfers. Ethiopia’s PSNP uses a combination of geographic and community-based targeting, with the transfer going to the household head. While it has been shown that community-based targeting effectively identifies the poor, geographic targeting that considers differences among homogenous poor and vulnerable groups remains questionable (25). Specifically, the inclusion of the most deprived and vulnerable people, harmonization with other interventions, and sensitization of all stakeholders on the role of women are far from optimal (26).

Generally, ensuring food and nutrition security at the household level requires investment in nutrition-sensitive interventions, protecting women’s rights, and improving their social and nutritional status (19). A key step in understanding the difference PSNP makes is to understand the relationship between poverty levels based on household consumption and the asset-based wealth index, because asset holdings are a critical outcome of PSNP (27). Based on this, an appraisal of the effect of PSNP across geographic areas is of great interest in terms of women’s nutritional status and the ability of their households to access an adequate quantity and quality of food to promote positive health outcomes. In addition, it is important to assess whether a combination of women’s nutritional status and household dietary diversity (HDD) predicts membership in PSNP and certain wealth groups. Therefore, this study was intended to assess factors associated with differences in HDD and women’s body mass index (BMI) among PSNP and non-PSNP households.

## Methods

A community-based cross-sectional study was carried out in the Kombolcha District of Eastern Ethiopia from July 1 to 28, 2015. This period overlapped with failed spring (mid-February to May) rain that affected crop production from the first harvest that would provide 20% of food production followed by the end of 6 months of PSNP cash transfer (28). The district contains 19 *kebeles* (smallest administrative units in Ethiopia next to districts), out of which 10 are non-beneficiary and 9 kebeles (total of 2,375 households) benefit from cash transfers. This translates to about 9,752 people who receive cash in exchange for participating in public works and 1,409 people with direct support. For this study, five PSNP and six non-PSNP kebeles were selected randomly, and only public works participants were included in the study.

Though fairness and transparency is the core principle of PSNP client selection, there are inclusion and exclusion errors. Corrupt officials, clan politics, and quota allocation were the main causes of inclusion and exclusion errors (22, 29). To obtain data with a low bias estimate, firstly, the data collection was carefully planned to include the same variables by using similar data collection tools and procedures for beneficiary and non-beneficiary households. Secondly, outcomes related to program participation were identified using key PSNP-related variables (livestock ownership, household landholding, access to government health post, asset depletion and food aid, and asset losses) that identify outcomes related to women’s nutrition and other related variables. This information was obtained from the *kebele* food security task force (KFSTF), which has seven members, including a health extension worker. Thirdly, to attain comparable access to market systems, similar livelihood zones known for khat and vegetable production were selected. These livelihood zones had similar agro-ecology and production patterns of these commercial crops; the participants had common livelihood strategies and comparable access to markets, including distance from the market. In this district, cash was provided because the markets functioned well.

Information about women was collected during the mother’s interview for eligible children aged 6 months to 5 years (information on children being processed in another publication). Hence, participants were selected from five randomly selected PSNP and six non-PSNP kebeles. Women eligible for a child interview were identified from lists obtained from the district PSNP office compiled by KFSTF and respective *kebele* health extension workers. Non-PSNP kebeles have similar KFSTFs that follow the same procedure to identify food insecure clients. Both PSNP and non-PSNP household lists are finally ascertained by social networks leaders called *gare* (groups containing 25–30 women). In order to minimize handout expectations and a spillover effect of the transfer, women from non-PSNP beneficiary households were entirely selected from non-beneficiary kebeles. Pregnant women and direct support beneficiaries were excluded from this study.

### Data collection procedures and quality assurance

A structured pretested questionnaire was used to assess socioeconomic and demographic characteristics of the households. Nursing students who could speak a local language (Afaan Oromo) were trained to collected data. The tool was pretested on 20 households to determine its suitability to local accent, format, wording, and order. In addition, periodic checking of the weighing scale and repeated measurement were used to assure the data quality.

Ethical clearance was obtained from the Haramaya University College of Health and Medical Science Institutional Health Research Ethics Review Committee. The objective of the study, known benefits, and risks of participant involvement in the research were communicated. Informed written and signed consent was obtained from women before commencing the study.

#### Variables

The primary outcome of this study was women’s BMI. The secondary outcome was Household Dietary Diversity Score (HDDS). In the statistical analyses, the factor considered as a potential confounder was maternal age. Factors considered as potential effect modifiers were the sex of head of household and PSNP beneficiary status.

#### Body mass index

BMI is a proxy indicator of energy status (undernutrition), calculated as weight (kg) divided by the square of height (m^2^). Women’s height was measured to the nearest 0.5 cm without shoes, feet flat, heels together, legs straight using a portable wooden height-measuring board with a sliding head bar following standard anthropometric techniques. Heights <145 cm were classified as stunted. Weight was measured repeatedly to the nearest 100 g using an electronic scale (SECA, Hamburg, Germany). A BMI of 17–18.4 indicates marginal energy deficiency, 16 to <17 moderate energy deficiency, and that of less than 16.0 indicates severe chronic energy deficiency. A BMI of ≥25 signifies overweight, and >30 signifies obesity. Even though a global database on women nutrition is not available, a BMI of 20–25 kg/m^2^ is recommended for good health and is associated with normal fertility. A weight for height equivalent to a BMI of 18 kg/m^2^ or lower is considered too low for successful reproductive ability (30).

#### Household Dietary Diversity Score

The HDD score is a measure of the total number of different food groups consumed in the last 24 hours by household members with a well-grounded construction of diet quality and accuracy, cross-checked with incomes. HDDS ranges from 0 to 12, the higher the better, and it is a good indicator of both quantity and quality. It is included in the acute food insecurity reference table for household group classification of the Integrated Food Security Phase Classification (IPC). HDDS does not have established categorical cutoffs and is analyzed only as a scale measure. A face-to-face interview was used to administer the tool. For households with unusual food intake in the previous 24 hours, another appointment was made for the interview. Due emphasis was placed on acquiring a response with minimal social desirability bias (31–33).

#### Household Wealth Index

Household wealth is a proxy measure of household income for long-term wealth. Principal components analysis was run using 38 items comprising productive assets, livestock, household goods, and consumer durables. It was used as a continuous variable, and each household was classified as being in the lowest, middle, or highest asset category.

#### Sample size determination

Analysis was performed on data that were already available for child wasting. Excluding 52 women, the final sample size was 623 women from PSNP and 635 non-PSNP (total 1,258). This sample size is sufficient for the analysis of the data to produce results with sufficient statistical precision.

### Statistical methods

Data were entered in EpiData 3.1 and the software package SPSS version 23 for Windows was used for statistical analysis. To examine whether associations differed across groups, stratification was done based on PSNP and wealth index. Descriptive statistical analysis was conducted to describe the characteristics of participants. For constructing wealth index based on 38 items, the selection of each factors was based on the rotated component matrix of greater than 0.5. One-way Analysis of Variance (ANOVA) was conducted. The independent-samples *t*-test was used to compare mean HDDS across PSNP and other variables. In order to check whether the assumptions of Multivariate analysis of variance (MANOVA) were met, preliminary assumption testing for normality, linearity, univariate and multivariate outliers, homogeneity of variance–covariance matrices, and multicollinearity were conducted. No significant violation was found. Further, an ordinal logistic regression model was used for prediction of women’s BMI (dependent variable). The odds ratio (OR) was used as the primary measure of strength and direction of the relationship between each independent variable and the women’s BMI values, which were categorized into underweight (BMI<18.4), normal (BMI 18.5–24.9), and overweight (BMI ≥25). In this analysis, OR less than 1 indicated a negative relationship.

## Result

The study included 1,311 women, of whom 39 were pregnant and 14 had out-of-range values, which resulted in a final sample size of 1,258. Table 1 shows the characteristics of participants stratified by PSNP membership, where 50.5% (653) were non-PSNP and 49.5% (623) were PSNP households. There were 146 (11.6%) female-headed households, mainly 57.5% (84) from PSNP households.

**Table 1:** Characteristics of women from PSNP and non-PSNP households in Kombolcha District of eastern Ethiopia, 2015

Variables	Non-PSNP (*n* = 635)	PSNP (*n* = 623)	Total (*n* = 1,258)
Head of Household			
Male	573 (90.2%)	542 (86.5%)	1,114 (88.4%)
Female	62 (9.8%)	84 (13.5%)	146 (11.6%)
Last pregnancy intentional			
No	100 (15.7%)	170 (27.3%)	270 (21.5%)
Yes	535 (84.3%)	435 (72.7%)	988 (78.5%)
Family planning use			
No	477 (75.1%)	422 (67.7%)	899 (71.5%)
Yes	158 (24.9%)	201 (32.3%)	359 (28.5%)
Breastfeeding now			
No	331 (52.1%)	371 (59.6%)	702 (55.8%)
Yes	304 (47.9%)	252 (40.4%)	556 (44.2%)
Less school attrition			
No	372 (58.6%)	293 (47%)	665 (52.9%)
Yes	263 (41.4%)	330 (53%)	593 (47.1%)
More health care services			
No	153 (24.1%)	172 (27.6%)	325 (25.8%)
Yes	482 (75.9%)	451 (72.4%)	933 (74.2%)
Reduced selling assets for food			
No	449 (70.7%)	519 (83.3%)	968 (76.9%)
Yes	186 (64.1%)	104 (16.7%)	290 (23.1%)
Vegetable garden ownership			
No	550 (86.6%)	546 (87.6%)	1,096 (87.1%)
Yes	85 (13.4%)	77 (12.3%)	162 (12.9%)
Wealth index			
Low	74 (11.7%)	341 (54.7%)	415 (33%)
Medium	196 (47%)	221 (35.5%)	417 (33.1%)
High	365 (50.5%)	61 (9.8%)	426 (33.9%)

**Fig. 1:**
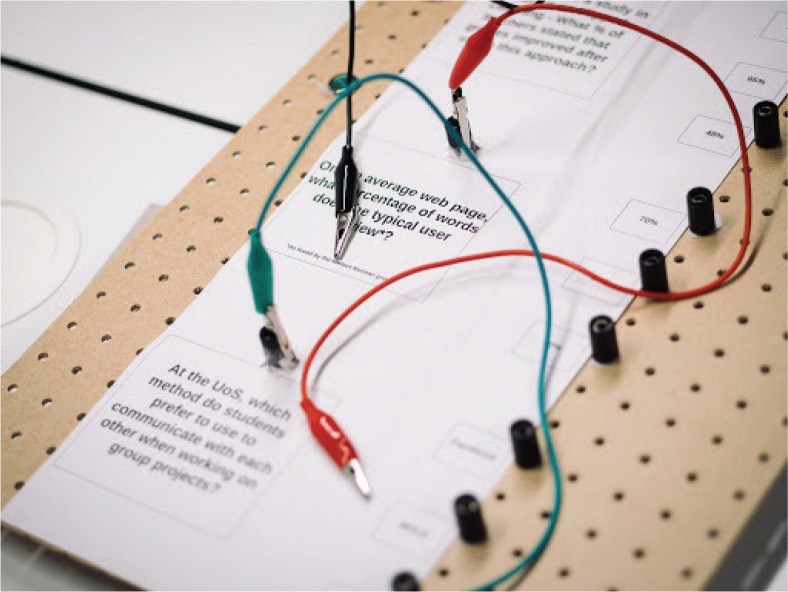
Prevalence of chronic energy deficiency of women from Productive Safety Net Programme (PSNP) and non-PSNP households in Kombolcha District, 2015.

The overall prevalence of underweight women (BMI <18.5) was 23.7% (95% confidence interval [CI]: 21.3–26.13). Out of this, the prevalence of BMI severe energy deficiency was 3.2% among PSNP households, which was higher than for non-PSNP households (1.4%) (Fig. 1).

The total mean HDDS was 5.76 ± 1.59 and the mean difference between PSNP and non-PSNP households was statistically significant. The magnitude of the differences in the means as indicated by eta squared was 0.142 (Table 2).

**Table 2:** Effect of PSNP on characteristics of women from PSNP and non-PSNP households in Kombolcha District, 2015

	Mean ± SD			Mean differences	Eta squared
	Total	Non-PSNP	PSNP		
HDD	5.76 ± 1.59	6.36 ± 1.5	5.15 ± 1.37	1.2**	0.142
Women’s BMI	20.29 ± 2.46	20.61 ± 2.46	19.97 ± 2.41	0.63**	0.017
Women’s age	28.75 ± 5.85	27.98 ± 5.64	29.54 ± 5.96	−1.6**	0.018
Number of children under five	1.73 ± 0.69	1.69 ± 6.80	1.76 ± 0.694	−0.066	0.002
Family size	6.2 ± 2.2	6.18 ± 2.43	6.23 ± 1.96	−0.047	0
Land size (ha)	0.56 ± 0.36	0.639 ± 0.412	0.470 ± 0.26	0.0194**	0.056

** *p* < 0.001. HDD, household dietary diversity.

A multivariate analysis of variance was performed to find any group differences based on a linear combination of women’s BMI that indicate utilization and HDD that showed the access and quality aspect of food insecurity. Inclusion of both dependent variables in the analyses provided the maximum amount of information regarding the effects of PSNP. Hence a two-way MANOVA was employed in which a 2 PSNP (beneficiary and non-beneficiary) ×3 wealth index (low, medium, and high socioeconomic status) were the between-participant factors. The combined dependent variable was significantly affected by state of PSNP membership, *F*(2, 1251) = 40.995, *p* < 0.001; Wilks’ lambda = 0.938, and the wealth index *F*(4, 2502) = 8.269, *p* < 0.001; Wilks’ lambda = 0.974, but not by their interaction *F*(4, 2504) = 2.2, *p* = 0.993, η^2^ = 0.004. The result showed association between PSNP and the combined dependent variable (η^2^ = 0.062) and wealth index (η^2^ = 0.013). This indicates that the linear composite of HDD and women’s BMI differs significantly with respect to PSNP membership and across wealth levels but not by their interaction (Table 3).

**Table 3:** Effect of PSNP, wealth index, and their interaction on women’s BMI and HDD for PSNP and non-PSNP households in Kombolcha District

Independent Variables	Dependent variables	Univariate *F*	*df*	Partial h^2^
Wealth index	Women’s BMI	2.9	2/1252	0.005
	HDD	13.70**	2/1252	0.021
PSNP	Women’s BMI	9.20**	1/1252	0.007
	HDD	75.90**	1/1252	0.057
Interaction of PSNP × wealth index	Women’s BMI	0.28	2/1252	0.000
	HDD	4.30*	2/1252	0.007

* *p* < 0.05

** *p* < 0.01.

Follow-up ANOVAs for investigating the main effect on the individual dependent variables (Table 4) indicated significant effects for PSNP both on women’s BMI and HDD. However, only HDD differed significantly across wealth levels. An inspection of the mean scores indicated that non-PSNP households reported significantly higher levels of HDD (*M* = 6.36, SD = 1.5) than PSNP households (*M* = 5.15, SD = 1.37). Similarly, women from non-PSNP households scored significantly higher BMI (*M* = 20.61, SD = 2.46) than women from PSNP households (*M* = 19.97, SD = 2.41), and PSNP membership had a stronger effect on HDD than women’s BMI (Table 2).

**Table 4:** Means and standard deviations of women’s BMI and HDD as a PSNP and wealth category for PSNP and non-PSNP households in Kombolcha District, 2015

Group	*n*	Women BMI		HDD	
		*M*	SD	*M*	SD
PSNP					
No	635	20.6	2.46	6.35	1.57
Yes	631	19.97	2.4	5.15	1.36
Wealth index					
Low	415	20.16	2.47	5.09	1.48
Medium	417	19.97	2.37	5.7	1.37
High	426	20.72	2.46	6.4	1.6

Tukey’s procedure to conduct pairwise comparisons of women’s BMI using an alpha of 0.01 for each outcome showed a significant mean difference in HDD across wealth categories (*p* < 0.001), with a more pronounced mean difference between low and high wealth levels. Hence, non-PSNP households scored higher on both HDD and women’s BMI (Table 4).

Generally, in the above two-way MANOVA, we asked if households along PSNP membership and wealth index were significantly different on a set of linearly combined variables (women’s BMI and HDD). The interaction effect of PSNP membership on combined dependent variables was not different across the wealth index gradient. Likewise, the effect of wealth index on dependent variables was similar for PSNP and non-PSNP households. Hence, the combination of women’s BMI and HDD did not predict PSNP or wealth group membership.

### Predictors of women’s nutritional status

To examine factors associated with nutritional status, women’s BMI measurements were categorized as underweight (BMI <18.5), normal (BMI 18.5–25), and overweight (BMI ≥25). Ordinal regression was conducted (Table 5) and yielded significant associations for wealth index, Uptake of better health care service compared to the previous year, and a reduction in selling assets for the sake of buying foods. As the most notable outcome, controlling for the other explanatory variables, women in the middle wealth index (OR = 0.533) had 46.7% lower odds than women from the high wealth index of being in the higher BMI category.

**Table 5:** Multivariable ordinal regression model for predicting the risk of higher category of BMI for women from PSNP and non-PSNP households in Kombolcha District, 2015

	B	SE(B)	*p*	OR	95% CI
HDD	0.067	0.065	0.307	1.069	0.941–1.215
Age of mother (years)	−0.008	0.020	0.665	0.992	0.954–1.030
Total land size (ha)	−0.240	0.221	0.279	0.787	0.510–1.214
Family size	0.057	0.049	0.240	1.059	0.962–1.166
PSNP					
No	0.165	0.218	0.450	1.179	0.769–1.808
Yes (ref)	0.000			1.000	
Wealth index					
Low	−0.343	0.269	0.203	0.710	0.419–1.203
medium	−0.629	0.231	0.006	0.533	0.339–0.837
High (ref)	0.000			1.000	
Sex of head of household					
Male	−0.190	0.306	0.530	0.825	0.453–1.504
Female (ref)	0.000			1	
Breastfeeding now					
No	−0.636	0.366	0.082	0.530	0.259–1.084
Yes (ref)	0.000			1.000	
Better healthcare services uptake^a^					
No	−0.436	0.209	0.037	0.647	0.429–0.974
Yes (ref)	0.000			1.000	
Less school attrition^b^					
No	−0.261	0.207	0.207	0.770	0.514–1.155
Yes (ref)	0.000			1.000	
Reduced selling assets for food^c^					
No	0.455	0.204	0.026	1.575	1.057–2.349
Yes (ref)	0.000			1.000	
Intention to have more children					
No	0.27	0.207	0.193	1.310	0.872–1.967
Yes (ref)	0.000			1.000	

a Household members’ health services uptake compared to previous year.

b School attrition of household members compared to previous year.

c Selling more assets to buy food compared to last year.

CI, confidence interval; OR, odds ratio.

## Discussion

This study was set out to assess the differences in women’s nutritional status as determined by BMI and the factors associated with it among PSNP and non-PSNP households. Emphasis was also given to understanding how an asset-based wealth index interacted with PSNP to influence HDD and women’s nutritional status.

Previous PSNP-related studies did not examine this issue. Hence, a lack of adequate literature was a challenge and points to the need for further studies in the future. On the other hand, this study is useful in informing PSNP stakeholders how to plan and allocate appropriate resources, especially for women. The study data were collected from PSNP and non-PSNP households only once and generalizability is limited to a similar population definition.

### Difference in women’s BMI and HDD

There were large differences in HDD with respect to PSNP, which was indicative evidence for an effect on household food access. This is consistent with findings from the Democratic Republic of Congo, where similar unconditional cash transfer recipients were free to spend the cash transfer for diverse reasons, and in particular for food items (34). However, the result is counter to evidence on vulnerability to drought and food price shocks in Ethiopia, which points to a greater risk of decreased consumption in drought-prone areas by non-PSNP households compared to PSNP households from price inflation (35).

However, contrary to expectation, PSNP has a minimal effect on women’s BMI. This mismatch might indicate the presence of an underlying factor that hinders translation of transfer into better nutrition outcome. Although it might be true that government initiated integration of nutrition into the PSNP late in 2016 (1, 36), the prevalence of underweight women is comparable to national figures. For instance, according to the 2011 EDHS reports, 27% of women were thin. This is comparable to PSNP (27.3%) but lower than non-PSNP (20.2%) household women. However, a figure of 6% overweight or obese is lower than the figure reported by EDHS (4). This contrasts with findings from rural India and Nigeria, where chronic energy deficiency is lower than obesity (37, 38). On the contrary, this study showed lower obesity, with an impending ‘food insecurity–obesity paradox’, with lack of recognition of the rising rates of overweight and obesity (36).

One notable finding was severe chronic energy deficiency for PSNP (3.2%) and non-PSNP (1.4%) women. Compared to findings from low- and middle-income countries that range between 1.8 and 6.2%, the PSNP result is comparable to Madagascar (3.4%). This level of undernutrition is related to high morbidity, mortality, and poor maternal–fetal outcomes with the potential of perpetuating intergenerational malnutrition (39). This reinforces how addressing conditional minimum preventative health care is mandatory (Brière & Rawlings, 2006). Evidence from Shigutes et al.’s (2013) report showed increased community-based health insurance uptake and retention through increasing demand and risk aversion behavior among PSNP households. This shows the untapped potential of PSNP for being a platform to address the most pressing maternal and child health care issues (40).

Like other similar social protection programs, there was a lower mean HDD among PSNP households that can be explained by the effect of cash transfers where markets are not able to respond to increased demand by increasing supply, thereby pushing up local prices and reducing access to food groups during usual lean transfer seasons (41). This finding is in favor of Sabates-Wheeler and Devereux (2010) work on Ethiopia’s PSNP that supports food transfers or a ‘cash plus food’ scheme as preferable to cash transfers alone in achieving the PSNP’s goal. This argument contradicts the cash-first policy of Ethiopia’s PSNP (1, 42).

The high magnitude of women’s undernutrition and lower HDD among women from non-PSNP is against the key design feature of a good public works program, which is inclusion of poor household as far as the wage rate is not higher than unskilled manual labor that do not attract households who are not poor (43). Taking into account PSNP membership and wealth index together, there was a difference in the mean of the linear combination of the HDD and women’s BMI, but with non-significant interaction. This demonstrates that the effect of economic status on the linear combination of the HDD and BMI is not different for PSNP and non-PSNP members.

From follow-up ANOVA, PSNP has a greater effect on HDD than women’s BMI. This means the program has a better outcome at household level than on women’s nutritional status. This might be a result of interaction within the household such as time use, money, and other resources that influence the nutritional status of women. This is consistent with the ‘collective approach’ intrahousehold resource allocation theory, which assumes transfer is influenced by the identity of the transfer recipient (man or woman), which in turn affects how this transfer is used and who benefits from it. To put it another way, 86.5% of PSNP beneficiaries are male-headed households and they are the recipients of the transfer. Hence, they influence how the transfer is used for household members as opposed to women recipients (woman-headed households), who tend to use them differently (44). Contrary to this, an analysis of the nutrition impact of Bolsa Família’s program found that household expenditure decisions in nutrition from increased bargaining power of women showed no evidence that favors this hypothesis, and impacts were driven by conditionalities that demanded households spend the transfer on health care and education, which recipients are not required to do in the PSNP (45).

### Predictors of women’s nutritional status

Women’s BMI results showed undernutrition because of energy deficiency, health status, and a lack of access to health services and sanitation. This study finding showed reduced BMI for middle wealth index women, a finding that aligns with other low- and middle-income countries where the highest wealth quantile is associated with better BMI (46). Nevertheless, for lower wealth index the BMI reduction was not significant. The most likely explanation for this finding is related to control, ownership, and the struggle to retain assets among the middle wealth group. It also raises concerns related to empowerment of women, which is positively associated with calorie availability and dietary diversity at the household level (47). This suggests the need for more attention not only to reproductive, social, and cultural norms but also differentials in income shocks and subsequent poverty (48).

Women from households that did not reduce sale of assets for food were 57.7% more likely to become underweight. A household’s distress sale of assets is one outcome indicator for the PSNP public works component in Ethiopia, and it shows the extent of change in a household’s short-term vulnerability to shocks. An Irreversible asset decapitalization and income shock reduce uptake of health care services, which subsequently leads to loss in labor productivity (49).

The analysis showed a higher proportion of breastfeeding women in PSNP households than non-PSNP households. Further analysis showed, though non-significant, lactating women had a higher risk of being in lower BMI category. This is congruent with the Ministry of Agriculture’s enhanced social assessment and consultation report that acknowledges health and safety risks for PLW participating in public works. As a result, PLW switch to temporary direct support (up to 1 year after birth) with co-responsibilities of attending health care (antenatal care, postpartum health facility visit and immunization) and nutrition services, such as growth monitoring and participation in nutrition-related behavioral change communication (1).

## Conclusion and recommendations

This study has provided evidence of the PSNP’s substantial effect on HDD and its minimal effect on women’s BMI. The overall mean value of women’s BMI was within the normal range. However, there was a high prevalence of female undernutrition and low HDD. The PSNP was born out of decades of emergency food aid that met only emergency nutritional requirements, with no lasting impact. Similarly, mainstreaming nutrition took three phases of PSNP to initiate. Hence, sooner or later strong behavioral interventions and other determinants of nutritional status must be addressed to unleash PSNP’s human capital development, especially on nutrition. These efforts should be supplemented through the proper implementation of income-generating opportunities and health-related soft conditionalities directed to women beyond a gender-based quota.

In addition, reassessing strategy to implement a rights-based framework to address chronically food-insecure households residing in non-beneficiary kebeles should be considered. The improvements noted in our study were not only revisiting women who were thought to be the primary target of this intervention, but also including comparative groups exclusively residing in non-beneficiary kebeles. However, the levels of empowerment of women, which is central to the success of the PSNP, need to be established well in future studies.
